# Elliott Thornton Glenny

**Published:** 1940

**Authors:** 


### ELLIOTT THORNTON GLENNY, M.B., B.S., M.R.C.S., L.R.C.P.

WE regret to record the death of Dr. E. T. Glenny on 11th February, 1940, at the early age of 59, after a distinguished career in many spheres and in various lands. Dr. Glenny was the son of Mr. and Mrs. Edward Glenny, of Barking, Essex, and was educated at Redland Hill House School, Bristol, and St. Bartholomew's Hospital, where he qualified in 1906 and became house-surgeon to Mr. Butlin. In 1908 he married Jessie, daughter of Mr. and Mrs. Dence, of Bromley, Kent, and with her he went in 1910 as a Medical Missionary in the Evangelical Union of South America to Cuzco in the Andes. After one year his wife died of typhoid fever, and he returned to England with his two daughters. Dr. Glenny then took part in two explorations, first with the Yale Harvard expedition to the land of the Incas in 1911, and in the following year up the Amazon and 500 miles of its tributaries to investigate the Putumayo rubber atrocities. During the war of 1914–18 Dr. Glenny served in France with the R.A.M.C., and proved to be an indefatigable and capable surgeon. He had early and extensive experience of gas casualties of all kinds whilst in France, and wrote some of his earliest reports on the effects of "poison gases" in the Field. After the war he returned to Bristol and practised for fourteen years in Clifton. In 1936 he retired from private practice to become Medical Instructor under the A.R.P. Department of the Home Office for the South Wales Area, and subsequently transferred to a similar post in the City of Bristol.

Glenny was a fine athlete who had represented his hospital and the three counties of Essex, Lincolnshire and Gloucestershire at hockey, and he was a member of the British Ski Club and a Fellow of the Royal Geographical Society.

He was a man of deep religious convictions, who in his student days had been secretary of the Christian Association at Bart's, and in his later days was chairman of the Bristol Medical Mission. Everything he took up was carried out enthusiastically and conscientiously.

In 1916 he married Winifred, daughter of Dr. Christopher Elliott, who, with the two daughters of his first marriage, survives him. To them we offer our deepest sympathy.

